# Critical, agentic and trans-media: Frameworks and findings from a foresight analysis exercise on audiences

**DOI:** 10.1177/0267323117737954

**Published:** 2017-11-02

**Authors:** Ranjana Das, Brita Ytre-Arne

**Affiliations:** University of Surrey, UK; University of Bergen, Norway

**Keywords:** Audiences, users, foresight, Internet of Things, IoT, future technologies

## Abstract

We write this article presenting frameworks and findings from an international network on audience research, as we stand 75 years from Herta Herzog’s classic investigation of radio listeners, published in Lazarsfeld and Stanton’s 1944 war edition of Radio Research. The article aims to contribute to and advance a rich strand of self-reflexive stock-taking and sorting of future research priorities within the transforming field of audience analysis, by drawing on the collective efforts of CEDAR – Consortium on Emerging Directions in Audience Research – a 14-country network (2015–2018) funded by the Arts and Humanities Research Council, United Kingdom, which conducted a foresight analysis exercise on developing current trends and future scenarios for audiences and audience research in the year 2030. First, we wish to present the blueprint of what we did and how we did it – by discussing the questions, contexts and frameworks for our project. We hope this is useful for anyone considering a foresight analysis task, an approach we present as an innovative and rigorous tool for assessing and understanding the future of a field. Second, we present findings from our analysis of pivotal transformations in the field and the future scenarios we constructed for audiences, as media technologies rapidly change with the arrival of the *Internet of Things* and changes on many levels occur in audience practices. These findings not only make sense of a transformative decade that we have just lived through but they present possibilities for the future, outlining areas for individual and collective intellectual commitment.

We write this article presenting frameworks and findings from an international network on audience research, as we stand 75 years from Herta Herzog’s (1944) classic investigation of radio listeners, published in Lazarsfeld and Stanton’s war edition of Radio Research.^1^ That study had significantly broadened the scope of inquiry, from a focus on media effects to research investigating audiences’ experiences, understood from their own perspectives and in the context of their everyday lives. As audience analysts have continued to explore meanings and uses of media, a crucial challenge lies in developing sound, scholarly and contemporary understandings of the fields in which we do our research. Shared by other fields in media and communications, this challenge deals with encompassing both academic developments and the shifting technological, cultural and social realities that constitute our research objects. Such debates are often conducted from the outset of a *temporal* axis, aiming to understand past developments bringing us to our present status and – even more challenging – predicting and suggesting future directions.

In the field of audience research, a significant body of work deals with recounting and critically debating the history and current contours of the field, and inspiring directions for future research (Carpentier et al., 2014; Livingstone, 2004; McQuail, 1997; Morley, 1992). This article aims to contribute to self-reflexive stock-taking and sorting of future research priorities, by drawing on the collective efforts of a new generation of European audience researchers. We write as directors of CEDAR – Consortium on Emerging Directions in Audience Research – a team of 29 audience researchers from 14 countries across Europe, funded (2015–2018) by the Arts and Humanities Research Council, United Kingdom, to conduct a foresight analysis exercise on developing current trends and future scenarios for audiences and audience research in the year 2030. The consortium first analysed emerging themes in the past decade, the findings from which were published as a special issue of *Participations* containing 13 articles (Das and Ytre-Arne, 2016). This work was followed by a foresight exercise comprising an analysis of trends in the field, a 14-country stakeholder consultation directed by colleagues David Mathieu and Miriam Stehling, and a horizon-scanning exercise to envisage the future of audience studies in 2030. This article presents frameworks and findings from CEDAR’s work.

The purpose of this article is, then, twofold. First, we discuss contexts and frameworks for our foresight analysis. Second, we present findings from our analysis of pivotal transformations in audience research and the horizon-scanning exercise we conducted for audiences. As media technologies rapidly change with the arrival of the *Internet of Things* (IoT) and change on many levels occur in audience practices, these findings could help make sense of a transformative decade that we have just lived through (Das, 2017), and present possibilities for the future, outlining areas for individual and collective intellectual commitment.

## Contexts of knowledge

To paint a picture of the context for this article, both long and short histories of audience research are necessary. The longer history of the field that we drew inspiration from considered the now 75-year history of interest in audiences, if we begin roughly around the time of Herta Herzog’s (1944) analysis of radio listeners. Paying close attention to this long history meant listening to the interdisciplinarity that always lay at the heart of a field which received contributions from literary theory (Iser, 1974; Radway, 1984), mass communications and sociology (Katz et al., 1973) and critical-cultural theory (Ang, 1985; Hall, 1980; Morley, 1980), amongst others – and paying attention to how different strands of theory have been prioritized by different voices within the field. It meant keeping in mind the premises behind active versus implied readers from film and print-mediated communication, and the attendant debates that these came with – around the over-celebration of divergence (Condit, 1989), or critiques about mis-readings of power from within political economy (Dahlgren, 1998), and carrying these critiques into newer interest in audiences and users. Staying grounded in this long history also meant scanning the boundaries of the field, noting where parallel work had gone on (Mathieu et al., 2016). We also returned, on occasion, to the seemingly dated but nonetheless relevant debates around administrative and critical research (Ang, 1987; Katz, 1987), as we engaged with stakeholders and developed horizon-scanning work. In this long history, audiences have been viewed as communities of interpretation (Fish, 1980), as agents as well as subjects (Ang, 1985), as publics and citizens (Livingstone and Lunt, 1994), as local, global and transnational, simultaneously fluid and located (Gillespie, 1995). Between these and further different understandings, there have been tensions, but also synergies and fruitful conversations, as made evident in the shorter history of audience research that we consider.

This shorter history spans *a transformative decade* (Das, 2017) for audience analysis, 2004–2014, which immediately preceded the inception of CEDAR. This decade was marked by scholarly curiosity and even uncertainty about the scope and premises of audience research, amidst attempts to argue for its continuing relevance (Bourdon, 2015; Livingstone and Das, 2013). Theoretical work involved attempts to bring audiences and users of interactive technologies together in a conceptual union (Livingstone, 2004), and dealing with understandings of audiences as produsers (Bird, 2011; Bruns and Schmidt, 2011). This decade was marked both by increasing uncertainty and even discomfort about foundational concepts to the field, such as reception or interpretation, and by the parallel recognition that these concepts must continue to work for us, albeit in refreshed ways, with doubts still remaining about users becoming ‘active participants’ (Van Dijck, 2009). As new media necessitated new modes of reading beyond the traditional conventions of print and audiovisual media (O’Neill and Hagen, 2010), the critical questions being asked in this decade were about the societal, cultural and democratic implications of these new modes of engagement (Carpentier et al., 2014; Nightingale, 2014). Markers of the questions these changes generated could be noted in articles, books, international projects and, as ever, in classrooms.^2^ CEDAR paid attention to this environment of curiosity by pursuing a detailed analysis of the transformations that caused conceptual questions around and about audiences to arise.

First, we asked, what had made the past decade so transformative for audience analysis, what, indeed, were these transformations? Second, how could we make use of these transformations to scan horizons and build an agenda for the field for the future? As will be made evident throughout this article, we found fruitful intersections between two key areas of interest in audience research: on one hand, technological transformations, anticipating the full arrival of the IoT (Ashton, 1999) as well as increasing concerns around privacy, trust and surveillance (Mansell, 2012), and on the other hand, shifting modes of political participation, in light of changing relationships between audiences and the state, public institutions, private and (semi)autonomous sectors. Aiming to understand the futures of audiences – and necessarily dealing with questions concerning people’s individual and collective practices in reference to power structures – a theoretically grounded understanding of these relationships was needed. While many potential routes were possible, we found inspiration in Anthony Giddens’ (1984) theory of structuration and conceptualized audiences as agents in dynamic relationships with diverse societal structures, seeking to create spaces for engagement and expression but also at times contributing to reproduce – willingly or not – the structures within which they operate. This became part of CEDAR’s developing framework, which we will now discuss.

## Framework and approach

**Figure 1.**
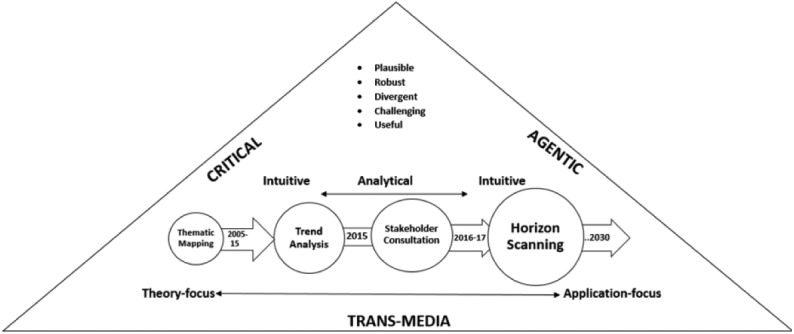
CEDAR’s CAT Framework.

CEDAR’s theoretical approach was defined by a framework where *Critical, Agentic, Trans-media* (CAT) (Figure 1) approaches underlie our conceptualizations of audiences and the priorities we adopted in scanning the future. The framework works in conjunction with other concepts taken from the long and short histories of audience research.

We begin with the C that stands for *Critical*. When embarking on a foresight analysis exercise, we were aware that much rich and informative foresight work came from industry and policy-focused projects. Some of these responded to urgent situations or even emergencies, many were feeding directly into policy or industry, many were commissioned to deliver measurable outcomes. As an academic network, it was necessary for us to recognize that the kind of horizon-scanning we would take up would often diverge from this. While we have adopted the methodology that lies behind horizon-scanning from practice- and policy-focused work, our approach has been critically academic. Keeping our eyes focused on this required CEDAR to emphasize the *critical*. Presenting our framework as a potential resource for other academics to engage in foresight work, we underline the importance of this. When we engaged with stakeholders in our 14 country-wide consultative exercise, we have been conscious of the value-laden baggage that the very term ‘stakeholder’ carries with it (Barker, 2014). Many of the terminologies we adopt with ease and criticism in academia – literacies, creativity and participation, for instance – are, with regularity, appropriated and co-opted within industry and even policy. To this end, we remained critical in two key ways when thinking about audiences as they often might be conceptualized within the industry. We paid close attention in our exercise with media industry stakeholders, to the discursive positions adopted by them in conceptualizing audiences (Mathieu et al., 2017). These positions revealed, disappointingly, that often audiences are held responsible for outcomes emerging from their engagement with intrusive technologies, thereby displacing responsibility from organizations and institutions. In parallel, concepts and findings in our trend analysis take an implicit or explicit critical view of commercial and technological power structures, as we discovered that audience creativity is often being co-opted and appropriated by industry (Stehling et al., in press), and creativity itself is becoming enmeshed within complex economic relations (Vesnic-Alujevic, Stehling et al., in press). When we developed work on interfaces and platforms, CEDAR has engaged with questions of ‘intrusive’ interfaces (Mollen and Dhaenens, in press), and further highlighted the often unequal dynamics of small and large flows of content between institutions and audiences (Pavlickova et al., in press).

The **A** of CEDAR’s CAT framework stands for Agentic. This – originating in the word agency – emerged at a network workshop where three parallel concepts – literacies, participation and creation – came together as CEDAR brainstormed its framework. Those arguing to place literacies within our framework emphasized centrality of making sense of people’s appropriation of newer technologies and affordances (Mollen et al., 2016; Ridder et al., 2016). Rather than taking a deterministic view of technology, the idea of literacies carries with it an interest in understanding audiences’ responses to, and potentially influencing, negotiating or shaping of, technological transformations. This discussion came with attendant concepts like capabilities, emergent from within economic theory (Sen, 2004), competencies, skills and its critical antipode so to speak with the idea of literacies (Livingstone, 2008), and was historically placed within media and communication studies’ long-term interests in the use and appropriation of media in everyday life (Bakardjieva, 2005). There was a parallel branch of theoretical interest in participation, including but not restricted to theories of democratic participation. Here, CEDAR’s mapping work showed a sustained interest in media and citizenship (Butsch, 2008; Carpentier, 2014; Dahlgren, 2009; Schrøder, 2012), reflecting the field’s long-term interest in democratic potentials and leading some to posit even the arrival of a new paradigm – the participatory paradigm in communication research (Livingstone, 2013). As Murru et al. (2016) noted, the key ‘*potential synergies or mutual obstacles between media and citizenship descend from the fact that they both produce and lean on some kinds of social entities in their basic processes*’ (p. 404) – all entities that CEDAR has been concerned with. Participation linked audiences as individuals and publics, with institutions in public life (e.g. Dahlgren, 2009; Lunt et al., 2014). Therefore, participation, alongside literacies, emerged as a strong component in our framework, and intersections – even tensions – between these components provide relevant starting points for investigating technological and political change in audiences

Finally, creation – encompassing but not restricted to the ideas of produsage (Bruns and Schmidt, 2011), small and large acts of content creation (Picone, 2011) and attendant questions of audience labour (Bird, 2011; Van Dijck, 2009) – occupied us. The network approached questions of creative participation in civic and cultural life (e.g. (Harrison and Barthel, 2009; Nakajima, 2012) and the educational and institutional implications of creativity within public policy (e.g. Recuber, 2012). What emerged out of the discussions about literacies, participation, creativity and their attendant concepts was that the words agencies and, by extension, agentic provided a space within which we could converge these articulations. However, this does not simply imply that we embrace the concept of agency as an umbrella for the others, but also that we refer to its key position in sociological theories of structuration, and of reflexive selves in conditions of high modernity (Giddens, 1984, 1991). As emphasized, agency constitutes a relevant theoretical conceptualization for capturing CEDAR’s interest in, for instance, co-option, audience labour and intrusive technologies – all referring to potentials and limitations in the power position of audiences as individuals and smaller groups, in relations to social structures and transformations.

Finally, the T of the CAT framework has stood for Trans-media. Very aware of the debates around the very words trans-media, cross-media (Lomborg and Mortensen, 2017; Schrøder, 2011), poly-media (Madianou, 2013) multiple literacies, the research on media repertoires (Hasebrink and Domeyer, 2012), CEDAR selected trans-media to represent and encompass the vibrant and busy conversation happening in the field along all these lines, including the divergences between them. Our approach was never bound by either genre or platform, and we adopted a very loose (on purpose) definition of audiences and *audiencing* (Fiske, 1992). The differences between parallel concepts of trans-media, cross-media and poly-media have not necessarily played an instrumental role in the way CEDAR has approached its work, but the centrality of blurred boundaries, diversely mediated texts and the rapid emergence of new genres has been central to our approach. Indeed, trans-media has kept ‘media’ central to CEDAR, not by placing the media in a box distinct from social and cultural life, but in keeping with the rich literatures on mediation (Livingstone, 2008; Silverstone, 2005) and mediatization (Lundby, 2014) which ensured that media production, regulation and audiencing, involving a constantly negotiated relationship (not always equal) between individuals/publics, industries and other institutions, was also central every time we thought through audiences on CEDAR’s exercises. Trans-media worked for our framework at multiple levels, from affordances and generic diversities to institutions and the ways in which audiences related with these. We note here, though, that we did not adopt the term as a ‘buzzword’ that signalled the redefining of all conceptual repertoires known to us. Rather, we drew from the T of the CAT framework, a reminder to pay attention to the media itself, its changing modes, norms, conventions, regulatory structures and discourses, in the context of new and emerging technologies, all of which invited us to reflect on the continuing value of concepts and terms across different kinds of communication conditions (Bolter and Grusin, 1996; Livingstone and Das, 2013).

## Methodology and design

The foresight analysis conducted by CEDAR found inspiration in three methods central to other foresight work – *trend analysis*, *stakeholder consultations* and *horizon-scanning* – but critically adapted them within the context of our developing framework. We cannot delve into the methodological detail for each of these here (for this, see Das and Ytre-Arne, 2017), but will instead focus on the overarching question of foresight as a relevant approach for assessing the future of a research field, reflecting on some merits and challenges of this approach. CEDAR was inspired by Van Notten’s (2006) account of the role of cultures of curiosity in foresight work – where these are defined as ‘*environments driven by inquisitiveness and imaginative thinking about the future. Such curiosity-driven research, free of vested interests and organizational impediments are likely to do more for free-thinking scenario development than any so-called scenario tool*’ (p. 88). This corresponds to our core idea of appropriating foresight analysis methods to the academic purpose of envisioning the future of a research field, applying such methods as starting points for scholarly debate rather than predictive mechanisms.

**Figure 2.**
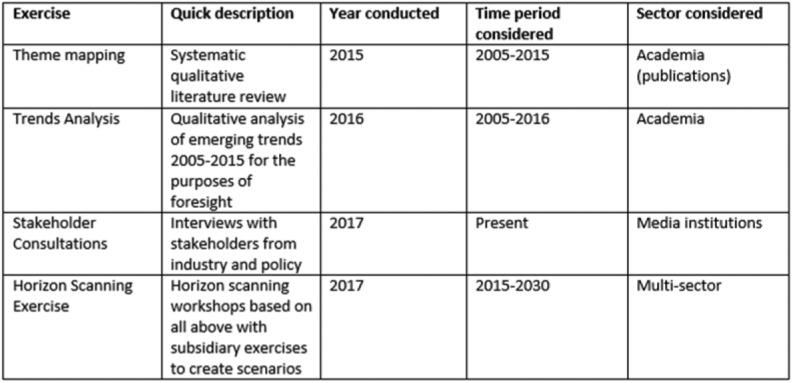
CEDAR’s methodology.

One of the first challenges we encountered early on was on defining the very scope and object of our inquiry, and working out the disciplinary boundaries we would seek our material within. As it stood in 2014 – the year that the funding bid for CEDAR was scripted – audience research could only be defined with great difficulty, for it had spread its roots among a variety of sub-fields and new fields in and outside media and communications, and yet, people continued to do (their own kind of) audience research. Constituted of researchers from some of these different disciplinary backgrounds, CEDAR could not easily pinpoint a singular definition of audiences to work from. This was a challenge, as we aimed to assess the status and future of a field with blurred conceptual boundaries. However, questions about the dis/continued validity of the ‘audience’ concept had perplexed the field in the decade or so preceding CEDAR, sitting against, but not referring to, a long-standing interest in the ‘retirement’ of concepts (Garrett, 2017; Kuhn, 1962). Instead of continuing this debate to find what would have been a less than satisfactory definition of audience analysis as a field, CEDAR (Figure 2) asked what kinds of transformations had started unfolding in the interface between new media technologies and their users and audiences, and what these transformations might say to us about the future.

In order to demonstrate our approach in the format of a journal article, we select key findings from two of the four exercises that CEDAR conducted: trend analysis and horizon scanning. These are closely interwoven, and serve to illustrate a temporal shift that is key to foresight analysis, from understanding the present towards developing future priorities. The next section focuses on outcomes of trend analysis – trends understood as key developments in the field as it stands currently, and after that, we follow these findings further into scenario development towards the future.

## Four pivots of transformation in audience analysis

The main aim of our trends analysis exercise was to distil pivots of transformations out of the diverse changes, on many levels, in the rich and vibrant decade that preceded CEDAR. From systematic mapping and thematic analysis of this decade (Das and Ytre-Arne, 2016), CEDAR developed and grouped micro-trends into four pivots of transformations. This shift, from a retrospective overview to a narrower focus on distilling the key trends we found to be continuing and growing in the here and the now, represents the first steps into foresight. Our analysis resulted in a focus on four pivots of transformations: (1) *audiences’ changing coping strategies with hyper-connected media*, (2) *audience interruptions of media content flows*, (3) *the co-option of audience labour*, and (4) *the micro–macro politics of audience action*. These are intended as pivotal points around which we might tell a broader story, and they are meant to speak on several levels. At a micro-level, each makes sense in its own right and helps us categorize and pinpoint developments we believe will rise in significance. At a meso-level, the pivots together tell a dialectic story that, in correspondence with the CAT framework, provides a critical account of negotiations in audience agency in a trans-media world. At a macro-level, this dialectic story helps us visualize audience agency in light of broader societal developments, a point to be taken up in our scenarios for the future.

The first pivotal transformation, *audiences’ changing coping strategies with hyper-connected media*, involves audiences’ reactions to the ubiquity and intrusion of media in their everyday lives and their multiple ways of dealing with this, including engaging with multiple screens, high levels of personalization of media and a variety of critical strategies that have been investigated in audience and literacies research but are taking on new significances in light of increasing intrusion and ubiquity of media. As Mollen and Dhaenens (in press) argue, producers increasingly construct restricted roles for users in algorithmic media, while audiences creatively appropriate and resist such ideal usages, pointing to the need to investigate rapidly developing sense-making strategies and ever newer literacies. This takes us to the second pivotal transformation, where we highlight small acts of audience engagement as *audience interruptions of content flows*. Especially when aggregated, everyday emanations of productive activity can interrupt the content produced by legacy media on a regular basis, forcing reactions from traditional productive agents. Pavlickova et al. (in press) discuss practices that transcend binaries between producers and audiences, ranging from commenting, debating and sharing through storytelling satire and re-configurations of content to activism or slacktivism. They suggest that interventions into societal discourses must be seen as a circular process, involving changing roles from audience to producer with many steps along the way, constantly re-configuring hegemonic relations between media-driven and audience-driven engagements.

The third pivotal transformation continues this thread through its critical interest in highlighting how *audience creativity is being co-opted by larger powers*. Vesnic-Alujevic, Stehling et al. (in press) highlight automated processes and algorithms as central to the design of digital platforms that allow for transforming audiences’ engagement into metrics. These technologies broaden the scale for how audience creativity can be taken up by industries and utilized for commercial purposes. Activities at the heart of this practice – such as user-generated content, citizen journalism or fan-fiction – can be positively framed as avenues for participation, but on an aggregate level, metrification and co-option can be seen as commodification, and even exploitation, of audiences’ free labour. This pivot touches upon critical questions concerning the agency of audiences, highlighting that relationships to media industries could be conflictive and that celebratory understandings of creativity need political and normative contextualization. This takes us to the fourth pivotal transformation, where we point at new kinds of audience engagements developing *between micro and macro politics of audience action*. Following up on the conflicting relationships that the three other pivots collectively portray of audience agency in complex power structures, we note the last decade’s key developments in terms of the political dimensions of audiences’ activities. Murru et al. (in press) discuss how audiences, in their everyday engagements with media, take up new possibilities to express narratives and form an action space where civic identities can evolve. Such micro-politics could be understood as a way of channelling emotions and literacies into social movements and organized collective action.

We find these pivotal transformations helpful in answering the question of what was transformative in the past decade in audience analysis. Continuing our interest in the agency of audiences within larger power structures, we now turn to how we envision the future for audiences through developing four scenarios for the future.

## Four scenarios for the future

In envisaging scenarios for the future of audiences, CEDAR has dealt with a short temporal frame of 1.5 decades and targeted the year 2030 to pinpoint our analysis. Our reasons for this choice concern a delicate balance between ambitions to be forward-looking and yet maintain clear connections to the present day. As we aim to formulate priorities and agendas for research, we have desired that these should be practical and realistically feasible.

### The scenarios as an analytical space

But a more critical point needs making at the very outset. The four scenarios we present are *not* intended to be a listing of what we think are four possibilities for the future. Indeed, countless scenarios might be created depending on what one is interested in studying. The four scenarios simply demarcate to us a combination of the four extremities of our long-standing interest in two dimensions (axes) that we have identified earlier in this article – (1) first, peoples’ diverse and divergent responses to emerging technologies including the IoT, and (2) second, shifting relationships between the state, commercial, (semi)autonomous institutions and audiences as individuals who participate in civil society. Because we snapshot the scenarios at the extremities of these dimensions, to open up and define the perimeters of an analytical space, the space is of greater essence than the extremities themselves. Because the four scenarios involve the extremities of these two axes, it would be misleading to read them as restrictive predictions – as though they predict that people will either engage with the IoT or not engage with the IoT, or that societies will either witness a large state or witness a small state, and similar such futile binaries. Instead, the scenarios open up and demarcate the *boundaries* of an analytical space within which we find the future in 2030 likely to unfold. It is critical to note, therefore, that rather than focusing scholarly attention on these four scenarios alone, or on pondering how likely these are, we might focus, more productively, on the space in-between, and consider the many possible interactions of these two dimensions we pursued as above, and the many changes that can be driven along these two dimensions (see Vesnic-Alujevic, Seddighi et al, in press) for a detailed account of the drivers of change in this context).

In making the choice to scan the horizon of what the contexts of audience research could look like in 2030, we note that we do this task at the brink of the potentially transformative IoT (Ashton, 1999) mediating the lives, worlds and practices of audiences as individuals and communities. We approach this with caution, not just because we cannot be certain of what degrees of enthusiasm about emerging technologies as societal and individual levels may or may not sustain itself into the future (cf. Borup et al., 2006), but equally because, as we write this piece, critical questions about media regulation, surveillance and privacy are beginning to overlap across conversations on social media, and those on the IoT (Mansell, 2012). We envisage cautiously that by 2030 we will have entered the high point of the IoT mediating most aspects of social, civic and political life in connected Europe – the context of our work. We work here with the concept of distance travelled between the early energy accompanying the appearance of a new form of mediated communication and its becoming ubiquitous in its uptake and developed in terms of the intellectual and socio-political critique around it. In doing so, far from being determined by technology, we follow the ways in which mediated experiences are likely to unfold over the foreseeable future. Therefore, comparing with the ubiquity of popular social media platforms and the distance travelled from the inception of these platforms till today, we envisage in 2030 that the IoT is widespread across Europe and increasingly integrated in daily life, and mediated experiences increasingly tailored to individual preferences and choices. In parallel, similar to what happened with the emergence of social networking platforms, we also anticipate that the intellectual critique of the IoT is reaching a state of maturity with well-developed theorizations of its social, cultural and political ramifications. It is this context that we position the four pivots of transformation presented previously, to hone in closely on their focus on (1) audiences’ interface with technological transformations and (2) transformations in public participation relating to the nature of relationships between public and private sectors and individuals. These two key axes have been long-standing interests in our conceptual framework, and we have seen them emerge out of our work on the four pivots of transformation, as discussed above, and they explicitly weave in and out of all the four pivots we presented. Surely, these were not the only axes we could have developed our scenarios around, and therefore, by extension, these are not the only possible scenarios. They remain, however, critical points of reflection, for us, as we scan the future, from the present.

### Four scenarios: The perimeters of an analytical space

**Figure 3.**
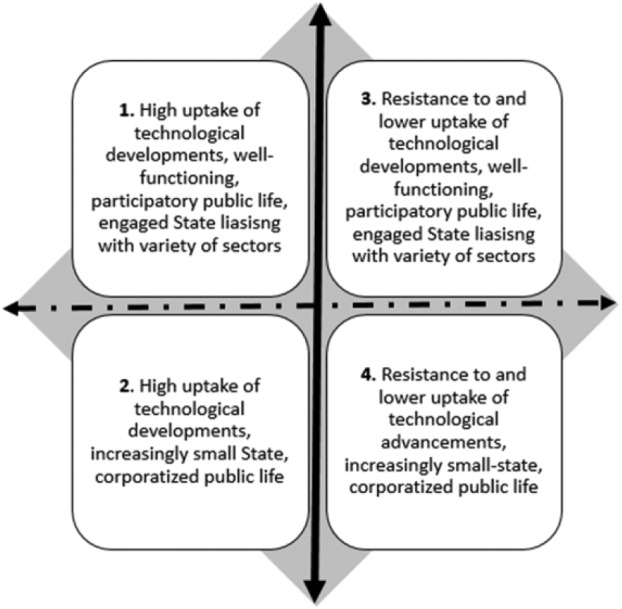
CEDAR’s scenarios as the boundaries of an analytical space.

In the graphic below, we see the bold broken arrow going horizontally, representing levels of public uptake and investment in the gamut of technological developments that unfold within, related to and outside of the IoT, including increasingly intrusive interfaces as developed by Mollen et al. (2016). We see the bold black arrow going vertically representing people’s participation in the public sphere, including the relationships of audiences as individual actors with institutions, both private and public. While we snapshot our scenarios (Figure 3) at two ends of this – (1) the social-democratic vision envisages a state involved with a variety of sectors participating in healthy public life, and (2) the more neo-liberal vision sees a small and receding state, corporatized public life and many commercial players dominating most aspects of public life. We present our scenarios as mirror scenarios – in pairs – each pair presenting two scenarios that paint opposite pictures along the axes above.

In one quadrant, we see in 2030 most individuals, households and organizations using connected gadgets which have complexified the IoT from a nascent stage today. From the projected 50 billion connected devices by 2020, there is a manifold increase in 2030, including diverse artefacts from all social arenas. We work with the idea here that automation has become less cumbersome, and more intrusive and subtly present in people’s lives. This is attendant with critical conversations on security and privacy, generally high levels of public awareness and critical literacies, and lively debates about data ownership, privacy, legalities of data, accessing of risky and harmful content by vulnerable audiences, surveillance and so forth. We tentatively see gaps in people’s access to this technological capital closing, as the IoT ends up being more ubiquitous, affordable and accessible. At this spectrum of the set of quadrants, we see a healthily functioning democracy with an engaged state involved with a variety of other sectors to promote education, health and emotional well-being. Technological transformations have had central roles to play in these sectors coming together to advance formal and informal literacies concerning media and technology. Social movements have become crucial avenues of participation in public life and are contributing in parallel to the healthily functioning democracy. A related scenario on the quadrant as shown above shares these facets around people’s engagement with mediated communication but departs from it in terms of the nature of public participation and the role of the state. This scenario sees people participating less in small acts of self-directed engagement with the media, and more in audience labour that is cleverly co-opted by larger and more powerful institutions. There is an increasingly neo-liberal public life with a small and receding State with diminishing involvements and regulatory responsibilities. We envisage here that technological transformations have had central roles to play in private sectors coming together in formal and informal education, media and technical literacies and education, healthcare and well-being. There is large-scale co-option of audience labour, corporate surveillance and exploitation of data. Both these scenarios converge in their visions of public engagement with the IoT in mediated societies of Europe, but they diverge, therefore, in the nature of public participation and institutional participation in the public sphere.

The contrasting pair of scenarios, as far as people’s engagement with technology is concerned, envisages that towards 2030 scepticism and critique of intrusive technologies have continued to rise unevenly across the population, but steadily nonetheless. Key concerns about intrusive, automated technologies, which were voiced in specific circles, entering the 2020s, have increased in complexity, having to do with the legalities of data ownership and protection, boundaries between public and private and increasing levels of surveillance that the IoT has fed into. As the IoT has burgeoned over the 2020s, significant pockets of resistance have developed that have refused to take up technological advancements as keenly. As a consequence, the population is increasingly fragmented between those who have chosen to resist and those who have not. There are some widening gaps also in terms of people’s access to technological capital.

We see two versions of this playing out depending on the nature of state–public relationships and the nature of public participation. In one, we envisage a healthily functioning democracy with an engaged state involved with a wide variety of other sectors, facilitating various forms of non-mediated public connection. In its other version, we see an increasingly neo-liberal public life with a small and receding State with diminishing involvements and regulatory responsibilities. Here, we witness the large-scale co-option of audience labour, corporate surveillance and exploitation of data, but with significant sections of the population escaping these by opting out of technical engagement. This however is only meaningful at an individual level, representing the eternal struggles between agency and structure. In opting out of technological developments which they find intrusive, we envisage that some may have also missed opportunities for participation and communication, and these gaps are affecting the development of newer literacies seeing a more uneven and fragmented field of technical and critical skills in the context of a highly privatized playing field. This might, then, have resulted in uneven conditions for small- and medium-scale social movements to become avenues of resistance in public life.

## Conclusion

These scenarios are intended to provoke thought, and anticipate the direction that research agenda might take within the field. They are intended to create an analytical space for discussing what the future may look like, a short while away from now. Coming out of a horizon-scanning exercise, they are *systematic projections* of *different combinations* of trends and developments along predefined axes. The scenarios are divergent and, by default, extremities of a space, but they are intended to be utilized as perimeter markers of a space within which scholarly concerns around the future of audiences might focus. Within the analytical space they create, scholars and stakeholders might evaluate how research and developments in media technologies, public life and a variety of other sectors can be placed and considered in relation to each other. This is particularly useful as we foresee burgeoning interest in the IoT and its implications in media and communications research, and anticipate substantial and highly diverse bodies of research to be published in the years to come. Similarly, with changing political conditions in Europe and resulting concerns over democratic participation in public life, the scenarios offer tools for considering the role of media and technology, highlighting both potentials and pitfalls.

In this article, we highlighted the significance of audience agency at the intersection of technological developments and changing political realities. This took us from our analysis of transformations in the last decade that also inform our understanding of where audiences and audience research stand today, towards scanning horizons for the future. Presenting pivotal transformations from the last decade, we emphasized four developments that come together in a dialectic story of how audience agency is negotiated – as media becomes increasingly ubiquitous, intrusive and hyper-connected; as new media technologies allow for creation and participation, and for co-option and exploitation of audience engagement; and as the micro–macro politics of audiences as actors become more complex. As these developments continue and grow in the future, taking on new meanings and dimensions with the IoT, we see great challenges and possibilities ahead for audience researchers. The analytical space created by the scenarios we have presented is populated with topics, examples and research questions that merit investigation from audience researchers and from scholars in many adjoining fields. Particularly, in the light of media intrusions as part of the unfolding IoT landscape, and in the light of the prospects identified in our foresight exercise, a logical next step is to think further about implications for audience agency in this new communicative environment.^3^ We particularly emphasize the continued need to critically evaluate intersections between technological and political transformations, and for doing so from the perspectives of individuals and social groups as actors within complex power structures.

## Notes

1. Of course, we concede that one could start dating the history even earlier with the Payne Fund studies on media effects, for instance, which we chose not to in this case as they did not sit comfortably with the newer, critical version of audience research. There are also single and little known studies here and there, such as Emilie Altenoloh (1913), on cinema audiences.

2. The Transforming Audiences conference series in the United Kingdom, the COST Transforming Audiences network, the beginnings of the journal participations and the changing module contents of audience modules across institutions were some of the markers of transformation in this transformative decade.

3. This point on agency is in discussion at the moment between the authors of this paper. The authors also wish to thank colleagues at the Lisbon Audiences2030 conference for their thoughts on this, and they thank Sonia Livingstone for her reminders to reflect on this in her critique of the CEDAR Final Report.
